# Signaling Responses to N Starvation: Focusing on Wheat and Filling the Putative Gaps With Findings Obtained in Other Plants. A Review

**DOI:** 10.3389/fpls.2021.656696

**Published:** 2021-05-31

**Authors:** Lingan Kong, Yunxiu Zhang, Wanying Du, Haiyong Xia, Shoujin Fan, Bin Zhang

**Affiliations:** ^1^Crop Research Institute, Shandong Academy of Agricultural Sciences, Jinan, China; ^2^College of Life Science, Shandong Normal University, Jinan, China

**Keywords:** microRNA, nitrogen starvation, phytohormone, root system architecture, signal, wheat

## Abstract

Wheat is one of the most important food crops worldwide. In recent decades, fertilizers, especially nitrogen (N), have been increasingly utilized to maximize wheat productivity. However, a large proportion of N is not used by plants and is in fact lost into the environment and causes serious environmental pollution. Therefore, achieving a low N optimum via efficient physiological and biochemical processes in wheat grown under low-N conditions is highly important for agricultural sustainability. Although N stress-related N capture in wheat has become a heavily researched subject, how this plant adapts and responds to N starvation has not been fully elucidated. This review summarizes the current knowledge on the signaling mechanisms activated in wheat plants in response to N starvation. Furthermore, we filled the putative gaps on this subject with findings obtained in other plants, primarily rice, maize, and *Arabidopsis*. Phytohormones have been determined to play essential roles in sensing environmental N starvation and transducing this signal into an adjustment of N transporters and phenotypic adaptation. The critical roles played by protein kinases and critical kinases and phosphatases, such as MAPK and PP2C, as well as the multifaceted functions of transcription factors, such as NF-Y, MYB, DOF, and WRKY, in regulating the expression levels of their target genes (proteins) for low-N tolerance are also discussed. Optimization of root system architecture (RSA) via root branching and thinning, improvement of N acquisition and assimilation, and fine-tuned autophagy are pivotal strategies by which plants respond to N starvation. In light of these findings, we attempted to construct regulatory networks for RSA modification and N uptake, transport, assimilation, and remobilization.

## Introduction

Nitrogen (N) is an essential macronutrient in plant growth and development, acting as the cellular constituent for a large number of molecules, including amino acids, nucleic acids, chlorophyll, and phytohormones. In recent decades, increasing the input of N fertilizers has contributed considerably to improving crop productivity. However, it is alarming to note that major cereal crops, such as wheat (*Triticum aestivum* L.), rice (*Oryza sativa* L.), and maize (*Zea mays* L.), only utilize 30–40% of the applied N. Apart from the unnecessary expense of wasted fertilizer, excessive input of N has also caused serious environmental problems and some negative effects on plant growth in certain cases. Therefore, improving the nitrogen use efficiency (NUE) of crops under suboptimal N conditions has emerged as an effective strategy to promote agricultural sustainability worldwide (Liu et al., 2018).

Plants can immediately sense nutrient availability, generate signal messengers, and translocate to the cell nucleus to induce genetic and downstream responses and thereby maintain nutrient homeostasis (Paul et al., 2015). To date, increasing numbers of investigations have been performed that have focused on understanding the physiological and biochemical mechanisms underlying the plant response to N starvation. Upon N stress, plants induce specific N signaling and construct signal transduction pathways, including biosynthesis of signal molecules, initiation of transcriptional modulation, and induction of the expression of regulatory and functional genes encoding N transporters and the main enzymes involved in N assimilation (Remans et al., 2006; Gojon et al., 2011; Wang et al., 2012, 2019b). These systematic signaling networks play important roles in the response to low-N stress (Wang et al., 2019b). Although a large number of investigations have been carried out, the sensing and perception of N starvation signaling and tolerance to low-N stress in plants have not been fully elucidated, especially in cereal crops (Jung et al., 2018; Liu et al., 2018; Qiao et al., 2018). Thus, first, we explored the signaling regulatory pathways that may be associated with morphological adaptation, N uptake and assimilation, and the corresponding biochemical processes. A better understanding of signal transduction for high NUE may establish a foundation for the molecular breeding of N starvation-tolerant crop cultivars.

Wheat is one of three most important cereal crops worldwide. This crop exhibits a dominant production of 772 Mt year^–1^, which is lower than 1135 Mt year^–1^ for maize but slightly higher than the 770 Mt year^–1^ for rice (FAO Stats 2017). These three crops represent approximately 90% of all cereal production worldwide (Ladha et al., 2016). Wheat production requires a large amount of N for both grain yield and quality (Zörb et al., 2018). Thus, timing and precise N application or using genetic means to improve the ability of wheat plants to grow well under suboptimal N are required for sustainable agriculture. Although most N-responsive genes are regulated by hormones and other signals, how wheat plants establish networks by which wheat tolerates or efficiently uses limited N has not been fully elucidated at the molecular, posttranscriptional, biochemical, and developmental levels.

The most important strategies for responding to N starvation include a number of pathways, such as signal generation, regulation of gene expression and metabolism, and morphological adaptation. In this review, we summarize the significant progress made in our understanding of environmental N sensing and signaling in recent years and discuss how low N signaling is integrated with other regulatory elements and regulates the expression of genes involved in the N uptake and assimilation pathways in wheat. Although many N sensing and regulatory components are borrowed from other species, such as *Arabidopsis thaliana*, rice, and maize, the components identified to date tend to have conserved functions and may be characterized by their orthologs in wheat. This review may help to elucidate the mechanism underlying which wheat responds to N starvation and benefits genetically engineered crop cultivars with improved NUE under low-N cultivation conditions.

## Calcium Messenger and Related Proteins

Calcium (Ca^2+^) is a ubiquitous secondary messenger and is widely involved in signal transduction pathways that regulate eukaryote responses to biotic and abiotic stresses. Ca^2+^ signaling is mediated by three sensor protein families, calmodulins (CaMs), calcineurin B-like proteins (CBLs), and calcium-dependent protein kinases (CDPKs), and its downstream signaling cascade includes kinases, such as CIPK8 and CIPK23, and TFs, such as ANR1, nodule inception-like protein (NLP) 7, and SPL9.

In rice, N starvation increases the leaf Ca^2+^ contents, especially in line with high tolerance to low-N stress (Tang et al., 2018). In particular, Ca^2+^ binding is an essential way to regulate the expression of genes in wheat roots that are associated with N stress responses (Zhang et al., 2016). In wheat, CaM, which can bind four calcium ions, is upregulated by low-N treatment and is involved in the promotion of primary root growth (Xu et al., 2019a). The CaM-related calcium sensor proteins, OsCML22 (predicted target gene of miR164) and Ca^2+^-transporting ATPases (predicted target gene of miR1318), are significantly upregulated in rice (Nischal et al., 2012), and transcripts of CaM, CaM-binding protein, and Ca^2+^-binding EF-hand protein are upregulated in maize roots under N starvation (Mascia et al., 2019).

CBLs can interact and activate CIPKs, forming the CBL-CIPK network as a component of the Ca^2+^ signaling pathway. CBL7 is upregulated and positively regulates the NO_3_^–^-dependent induction of *NRT2.4* and *NRT2.5* gene expression under NO_3_^–^ deprivation (Ma et al., 2015; Sharma et al., 2017). *CIPK8* acts as a positive regulator in nitrate low-affinity responses under low-N stress and is involved in long-term nitrate-modulated primary root growth (Hu et al., 2009). CIPK23, which is activated by CBL1 and CBL9 and is dephosphorylated by ABI2 (a member of the PP2C family), phosphorylates NRT1.1 under low-NO_3_^–^ conditions, thereby converting it from a low- to high-affinity transporter (Sharma et al., 2017). In wheat, both *TaCIPK8* and *TaCIPK23* are upregulated under low-N conditions (Mahmoud et al., 2020). Overexpressing *OsCIPK2* increases Ca^2+^ uptake, with plants exhibiting higher NO_3_^–^ uptake and root and shoot growth in rice under low N stress (Khan et al., 2019). Taken together, these findings suggest that Ca^2+^ signaling pathways may be involved in a regulatory cascade for metabolic processes, including modification of root architecture, N uptake, and transport under N starvation, mainly via reversible protein phosphorylation.

## Transcription Factors: Multiple Mediators of the N-Starvation Response

**FIGURE 1:**
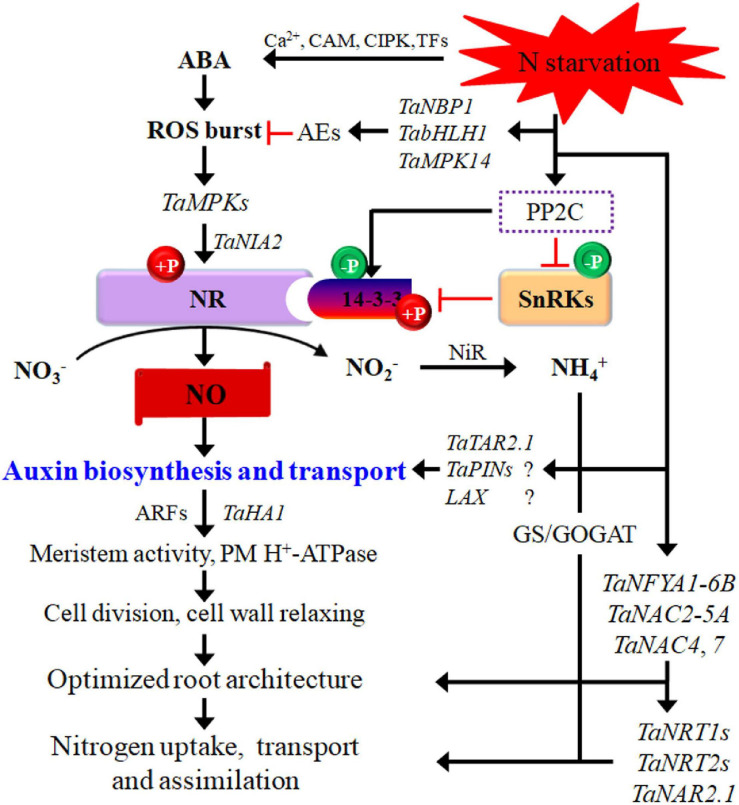
Potential pathways regulating wheat responses to low-N stress. N starvation interacts with phytohormones signaling. The interactions between them regulate root cell activity via ARFs and thus modify the root system architecture. Black arrows indicate positive regulation of N transport or assimilatory pathways, and red lines with short vertical columns indicate negative signaling steps. AEs, antioxidant enzymes; ARF, auxin responsive factors; bHLH, basic helix–loop–helix; GOGAT, glutamate synthase; GS, glutamine synthetase; MPK, mitogen-activated protein kinase; NBP, nucleotide-binding protein; NFYA, nuclear factor Y subunit A; NR, nitrate reductase; SnRKs, SNF1-related protein kinases; TAR, tryptophan aminotransferase-related. Question marks in the figure indicate that the important components in low-N signaling networks remain to be elucidated in wheat.

An increasing number of TFs have been identified to be involved in the N-starvation response via transcriptional modulation of numerous N-responsive genes. TFs may be important signaling components and can modify root plasticity, increase N uptake, and promote N metabolism (Table 1). NF-Y TFs are induced or upregulated in the roots and shoots of wheat seedlings by low-N stress, increasing N uptake and grain yield (Qu et al., 2015; Curci et al., 2017). TaNFYA1-6B is a low nutrient-inducible TF on chromosome 6B that encodes one of the subunits of NF-Ys. TaNAC2-5A is a cereal-specific NAC (no apical meristem protein, Arabidopsis transcription activation factor, and CUp-shaped cotyledon) TF and can directly bind to the promoter regions of the genes *TaNRT2.1-B1*, *TaNPF7.1-D1*, and *TaGS2-2A*. Overexpression of *TaNFYA1-6B* and *TaNAC2-5A* TFs significantly stimulates lateral branching, enhances the expression of *NRT1* and *NRT2* families, and positively regulates *TaGS2* expression and root growth, thereby promoting N uptake, N assimilation, tiller numbers, spikelet number, and grain yield in wheat under low-N conditions (He et al., 2015; Qu et al., 2015; Figure 1).

**TABLE 1:** Transcript factors in response to N starvation.

TFs	Regulated genes	Species/tissues	Putative function	References
TaNFYA1-6B and TaNAC2-5A	*TaNRT2.1-B1*, *TaNPF7.1-D1*, and *TaGS2-2A*	Wheat roots and shoots (upregulated)	Stimulating lateral branching, increasing N uptake and grain yield	He et al., 2015; Qu et al., 2015; Curci et al., 2017
MYB	*NRT2.1*, *NRT2.2*, *NAR2.1*, and *OsNiR2*	Durum wheat roots and leaves/stems (up- or downregulated), wheat leaf (downregulated), wheat root (upregulated)	Regulating cell development and the cell cycle, increasing N influx, the tiller number, shoot dry weight, and total N content	Zhang et al., 2012; Curci et al., 2017; Wang et al., 2019b; Wang et al., 2020a
bZIP	*TaNRT2.1*, *TaNADH-GOGAT*	*TabZIP60*: wheat root (upregulated), *TabZIP1* (an ortholog of *AtbZIP16*): wheat root and shoot (upregulated), *bZIP77* and *bZIP53*: maize root (upregulated)	ABA signaling, negatively regulating lateral root branching, N uptake, NADH-GOGAT activity, spike number, and grain yield	Yang et al., 2019a; Ma et al., 2020; Mahmoud et al., 2020
TaZFP593;l and TaZFP15	*NtPIN3*, *NtNRT1.2-t*, *NtNRT2.2*, *NtCAT1*, *NtCAT3*, *NtPOD1;1, NtPOD1;7, NtPOD2;1* and *NtPOD9*	Wheat leaf and/or root (upregulated)	Mediating RSA establishment, N acquisition, and cellular ROS homeostasis; low-N signal transduction	Sun et al., 2012; Chen et al., 2017
WRKY		Wheat root (upregulated), durum wheat root (most downregulated in N chronic stress)	Establishment of complex signaling networks to increase N stress tolerance	Curci et al., 2017; Wang et al., 2019b
DOF	Genes encoding enzymes for carbon skeleton production, amino acid synthesis and a reduction in the glucose level; *AMT1*, *AMT2*, and *AMT3*, *PEPC*	Durum wheat root (upregulated)	Regulating external nitrate response and internal N assimilation promoting NH_4_^+^ uptake in roots	Yanagisawa et al., 2004; Kurai et al., 2011; Curci et al., 2017; Wu et al., 2017
TaNLP	*NRT1*.*1* and auxin signaling F-box (*AFB3*)	Wheat root and/or leaf (upregulated or induced)	Involved in metabolic and regulatory processes associated with NUE and nitrate signal transduction pathway by binding to the nitrate-responsive elements	Castaings et al., 2009; Konishi and Yanagisawa, 2013; Yu et al., 2016; Kumar et al., 2018

Most MYB genes are differentially (up- or downregulated) expressed in roots and leaves/stems of durum wheat under N starvation (Curci et al., 2017). In wheat, the expression of genes for MYB78 in leaves is significantly downregulated, while MYB33 in roots is significantly upregulated under low-N stress (Wang et al., 2019b). The newly identified miRNA ttu-novel-106 is immediately and strongly downregulated in roots after N stress and is negatively correlated with the expression of its putative target gene *MYB-A* (Zuluaga et al., 2018). These results suggest that the ttu-novel-106/TtMYB-A coupling in response to low-N stress may contribute to the adaptation or resistance of plants to low-N conditions. In the roots of *OsMYB305*-overexpressing rice lines, the expression of *OsNRT2.1*, *OsNRT2.2*, *OsNAR2.1*, and *OsNiR2* is upregulated, and ^15^NO_3_^–^ influx, tiller number, shoot dry weight, and total N content are significantly increased under low-N conditions (Wang et al., 2020a). MYB TFs are associated with cell development and the cell cycle in wheat under low-N stress (Zhang et al., 2012).

The MYB-related TFs bZIP77 and bZIP53 encoded by *MRP2* are upregulated by low-N stress and may play a positive regulatory role in maize root growth under low-N stress (Ma et al., 2020). *TabZIP60* is a wheat ABRE (ABA-responsive elements)-binding factor (ABF)-like leucine zipper TF; knockdown of *TabZIP60* through RNAi promotes lateral root branching, increases N uptake, NADH-dependent glutamate synthase (NADH-GOGAT) activity, and spike number and improves grain yield under field conditions, while overexpression of *TabZIP60-6D* has shown the opposite effects (Yang et al., 2019a), indicating that this TF acts as a negative regulator of the NO_3_^–^ response. Recently, *TabZIP1* (an ortholog of *AtbZIP16*) and *TaPIMP1* TFs have been found to be upregulated in both roots and shoots and identified as new players in the low-N response in wheat (Mahmoud et al., 2020).

*TaZFP593;l* is a C2H2-type gene encoding ZFP in wheat that bears a conserved C2H2 motif and targets the nucleus after sorting from the ER. *TaZFP593;l* plays a pivotal role in mediating RSA establishment, N acquisition, and cellular ROS homeostasis by transcriptionally regulating the genes associated with N starvation adaptation in wheat (Chen et al., 2017). Similarly, the transcription of *TaZFP15* is induced in wheat roots by N deficiency, suggesting that *TaZFP15* is involved in mediating low-N signal transduction (Sun et al., 2012).

WRKY genes have been reported to be signaling genes that respond to abiotic and biotic stresses, including low-N stress. The expression levels of WRKY TF genes are significantly upregulated in the roots of wheat under low-N stress (Wang et al., 2019b). However, most identified members of the WRKY family are downregulated in the roots of durum wheat in response to N chronic stress (Curci et al., 2017). Although there is still no direct evidence linking them to other components in regulatory networks, WRKY proteins may contribute to the establishment of complex signaling networks and thereby increase the N stress tolerance of plants (Curci et al., 2017).

DOF (DNA-binding with one finger) TFs are involved in various biological processes, including hormone signaling (Noguero et al., 2013). The DOF TF-encoding genes, particularly *DOF1*, act as crucial mediators of N starvation tolerance by regulating the external nitrate response and internal N assimilation (Yanagisawa et al., 2004). Expression of the *DOF1.3* gene is upregulated in the roots of durum wheat plants when exposed to N starvation, and C2C2-Dof TFs are also upregulated in roots and shoot tissues (Curci et al., 2017).

In rice, the *OsDOF18* gene can induce ammonium transporter family members (AMT1, AMT2, and AMT3), thereby promoting NH_4_^+^ uptake in roots (Wu et al., 2017). Transgenic expression of the *ZmDOF1* gene increases N assimilation and plant growth under low-N conditions (Kurai et al., 2011). Furthermore, the expression of EcDof1 is accompanied by the expression of NR, GS and GOGAT in finger millet (*Eleusine coracana* L.), indicating that Dof1 probably regulates the expression of these genes (Gupta et al., 2009). On the other hand, overexpressing phosphoenolpyruvate carboxylase (PEPC) confers low-N tolerance and a higher grain yield by increasing carbon levels under low-N conditions (Tang et al., 2018). Overexpression of *ZmDof1* in *Arabidopsis* increases the expression of *PEPC* and several genes involved in the tricarboxylic acid cycle, thereby producing more carbon skeletons for the assimilation of N (Yanagisawa et al., 2004). This view is strongly supported by the finding in rice that TFs increase the net photosynthesis rate and carbon flow toward N assimilation and thus improve N assimilation and plant growth under low-N conditions (Kurai et al., 2011).

RWP-RKs represent a small family of TFs that are unique to plants and function particularly under N starvation conditions. NLPs (NIN-like proteins, one subfamily of RWP-RKs) have been confirmed to regulate the tissue-specific expression of genes involved in NUE (Konishi and Yanagisawa, 2013; Chardin et al., 2014; Kumar et al., 2018). The expression of two *TaNLP* genes (*TaNLP1* and *TaNLP2*) is induced by low-N conditions in mature leaves and is implicated in metabolic and regulatory processes associated with NUE (Konishi and Yanagisawa, 2013). The *NLP7* gene (Kumar et al., 2018) and *TaNLP4* (Mahmoud et al., 2020) are significantly upregulated in wheat under N-limited conditions. The *TaNLP7* gene has already been characterized in *Arabidopsis* and wheat and regulates the expression of *NRT1*.*1* and auxin signaling F-box (*AFB3*) genes (Castaings et al., 2009; Yu et al., 2016; Kumar et al., 2018). In wheat, NLPs may be involved in the nitrate signal transduction pathway, probably by functioning through their N-terminal regions that can bind specifically to nitrate-responsive elements (Kumar et al., 2018). In maize, TF ZmNLP6 is involved in modulating the early N response and root architecture through the mechanism of alternative splicing, rather than altering transcriptional abundance (Wang et al., 2020d).

## Multifaced Roles of miRNA in Response to N Starvation

**FIGURE 2:**
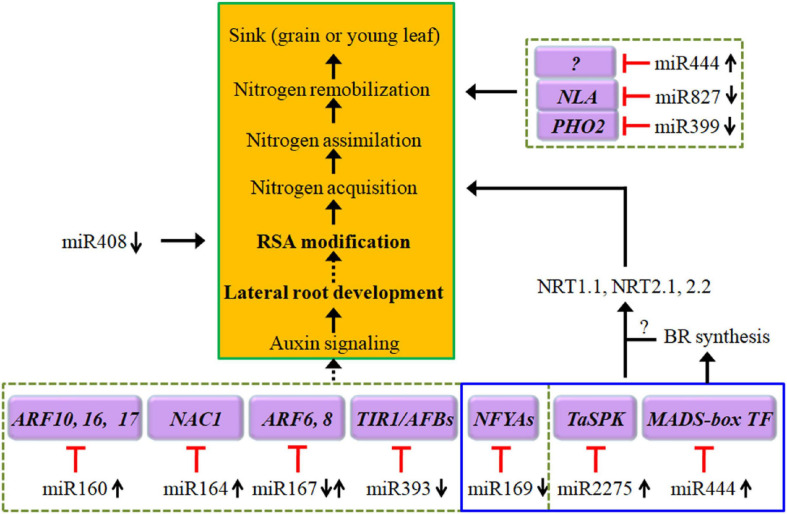
Diagram of low-N stress-induced alteration in the expression of a set of miRNAs that in turn regulate the expression of their targeted genes and then construct several regulatory pathways, including auxin signaling for root architecture modifications, expression of NRTs for N uptake and transport, and expression of NLA (nitrogen limitation adaptation) and PHO2 for N remobilization. The black arrows indicate positive signaling steps, the red lines with short vertical columns indicate negative signaling steps, and the dashed lines with solid arrowheads indicate putative regulation. See the text for further explanation. NFYA, nuclear factor Y, subunit A.

Plant miRNAs are highly conserved non-coding small RNAs that are involved in regulating cell signaling mechanisms, plant growth, and development by depressing gene expression at the posttranscriptional level or via translational inhibition. The expression of miRNAs can be induced or suppressed by N deprivation (Paul et al., 2015). To date, a large number of miRNA families have been identified in the transcriptional response to N starvation in wheat (Zhao et al., 2013; Sinha et al., 2015; Gao et al., 2016). The interactions between low N-responsive miRNAs and their target genes may establish distinct miRNA-mediated pathways for regulating the plant response to N deprivation (Zhao et al., 2015; Figure 2 and Appendix Table 1). miRNA-guided regulatory networks should provide new tools for the genetic improvement of wheat NUE. However, the mechanisms underlying the miRNA-modulated adaptation to N starvation remain to be further characterized due to the complexity of modeling the miRNA-mediated stress response.

### miR1118, miR1129, miR1133, and miR1136

In the roots of wheat, miR1118 is N-responsive and plays a role in RSA development under N starvation (Sinha et al., 2015; Zhao et al., 2015). In addition to TamiR1118, TamiR1129 and TamiR1136 are upregulated, whereas TamiR1133 is downregulated in roots under N-deprived conditions (Zhao et al., 2015). The target genes of these miRNAs are listed in Appendix Table 1. Notably, some circRNAs are involved in the regulation of low-N-promoted root growth, and TamiR1118 and TamiR1133 can interact with circRNA2473 and thus regulate root elongation (Xu et al., 2019b).

### miR159

miR159 is depressed in the grains of wheat under low N conditions (Hou et al., 2020). In root tissues, miR159a and miR159b are differentially expressed in response to N availability (Sinha et al., 2015). The N-responsive expression of miR159 has also been observed in the roots of maize (Zhao et al., 2012). Li et al. (2016) suggested that miR159 may function as a regulator of MYB and Teosinte branched1/cycloidea/proliferating cell factor (TCP) TFs and is associated with GA signaling by regulating the target *GAMYB* genes, thus influencing plant development.

### miR160

In *Arabidopsis*, overexpression of miR160 promotes lateral root development, resulting in more lateral roots under N-limited conditions (Gifford et al., 2008; Liang et al., 2012). The miR160/ARF17 module may function in RSA formation through the auxin-related regulatory pathway (Li et al., 2016; Figure 2). In durum wheat, ttu-miR160 tends to be upregulated, while *ARF22* (the ortholog of a *T. aestivum* gene similar to the *Oryza sativa ARF22*) is significantly downregulated in roots (Zuluaga et al., 2018). These results imply that N deficiency-induced expression of miR160, in combination with decreased ARFs, promotes lateral root production to access more N (Guo et al., 2005; Gifford et al., 2008; Liang et al., 2012). However, the expression of miR160 is only upregulated in roots in response to transient low-N conditions in maize (Xu et al., 2011) but is repressed by chronic N stress in wheat roots (Sinha et al., 2015). Moreover, several members of miR160 are downregulated in roots and shoots in rice (Shin et al., 2018) under both short-term and long-term low-N stress conditions. Therefore, the distinct roles of miR160/ARFs in response to low-N stress have not been clarified. In any case, some evidence has shown that overexpression of miR160 enhances lateral root development under N-limited conditions, which is believed to improve or boost the capability of plants to maximize the uptake of limited N availability (Gifford et al., 2008).

### miR164 and miR167

miR164 targets and cleaves the transcripts of *NAC1* (a NAC domain-containing gene); this miR164/NAC module may function in the production of more lateral roots through the auxin-related regulatory pathway because *NAC1* transduces auxin signals for lateral root emergence (Guo et al., 2005; Li et al., 2016; Figure 2). In the currently available literature, reduced expression of miR164 has been observed in wheat under chronic N stress (Sinha et al., 2015) and in durum wheat in short-term N stress, where ttu-miR164d is repressed corresponded to an upregulation of *NAC7* (an ortholog of *T. aestivum NAC7*) gene (Zuluaga et al., 2018), which may be consistent with the findings in *Arabidopsis* that plants with downregulated expression of miR164 produce more lateral roots due to its role in regulating the expression of NAC TFs (Guo et al., 2005). miR164 can interact with another predicted target of ubiquitin family proteins that is involved in protein degradation and remobilization, thus conferring plants with high low-N tolerance (Nischal et al., 2012).

miR164 and miR167 are coordinately responsive to N deficiency in wheat, *Arabidopsis*, maize and rice. Decreased expressions of both miR164s and miR167s are closely associated with low-N tolerance in rice (Nischal et al., 2012). In *Arabidopsis*, miR164 is upregulated (Guo et al., 2005), but miR167 is downregulated (Liang et al., 2012; Paul et al., 2015) under N starvation conditions. Similarly, Xu et al. (2011) found that under long-term low-N conditions, miR164 is upregulated in leaves, while miR167 is downregulated in the roots of maize. Once again, in roots of durum wheat with a higher NUE, miR164d is strongly upregulated; conversely, miR167h is significantly downregulated under N stress (Zuluaga et al., 2017). These results suggest that a spatial miR164/miR167 complex may be responsible for specific RSA adaptation to low-N stress (Zuluaga et al., 2017). The downregulation of miR167 by N starvation positively regulates adventitious root growth via a higher expression of its target genes *ARF6* and *ARF8* in roots (Xu et al., 2011; Liang et al., 2012); the latter is induced in pericycle and lateral root cap cells under N-limiting conditions as the regulator of lateral roots (Gifford et al., 2008). However, the opposite results have also been observed; N starvation induces the expression of miR167a and represses the expression of *ARF8*, which positively mediates lateral root initiation in *Arabidopsis*, indicating that *ARF6* and *ARF8* are associated with the repression of lateral root development during NO_3_^–^ limitation (Gifford et al., 2008; Figure 2). In the shoots and roots of maize, the expression of miR167s has also been reported to be upregulated by N deficiency (Zhao et al., 2012).

### miR169

Relative to other miRNAs, such as miR160, miR164, and miR167, the expression of miR169 is consistently repressed in the grain of wheat (Hou et al., 2020) and in the roots and/or shoots of *Arabidopsis*, rice, and maize upon N starvation (Xu et al., 2011; Liang et al., 2012; Trevisan et al., 2012b; Zhao et al., 2012; Qu et al., 2015; Yang et al., 2019b). The transcriptional repression of miR169s upon NO_3_^–^ starvation is a crucial step in integrating nitrate signals into metabolic, physiological, and morphological adaptations in maize roots (Trevisan et al., 2012b). Importantly, miR169 may function as a potential long-distance signal to regulate N starvation responses because a sharp decrease in miR169 in phloem sap has been observed in oilseed rape (*Brassica napus*) under N-limiting conditions (Pant et al., 2009). This long distance signaling occurring through the vasculature may play a fundamental role in maintaining nutrient homeostasis across the whole plant.

In wheat and rice under low-N stress, the miR169 family is downregulated, while nuclear factor Y subunit A (NFYA), the target of miR169, is upregulated (Zhao et al., 2011; Qu et al., 2015; Shin et al., 2018). In contrast, the constitutive expression of miR169a in transgenic *Arabidopsis* suppresses the accumulation of *NFYA* family members and decreases the expression of *AtNRT1.1* and *AtNRT2.1*, and transgenic plants accumulate less N and are more sensitive to N stress than wild-type plants, which may be attributed to impaired N uptake systems (Zhao et al., 2011; Figure 2). Overexpressing *TaNFYA-B1*, a low-N-inducible NFYA TF, significantly increases N uptake and grain yield, likely by upregulating auxin biosynthetic genes and the expression of N transporters in the roots and by stimulating root development (Qu et al., 2015). Furthermore, determination of the biological function of these targets using gene ontology (GO) analysis revealed that miR169/targets may also be involved in the regulation of N metabolism (Yang et al., 2019b). These results suggest that miR169 and *NFYA* family members may control a complex regulatory network.

### miR2275

Target genes of wheat TamiR2275 encode proteins involved in various biological processes, including signal transduction, transcriptional regulation, and stress response. miR2275 is gradually upregulated upon N starvation and thus positively regulates plant tolerance to low-N stress through transcriptional regulation of target genes, conferring plants improved N acquisition, better phenotype, increased biomass, and enhanced photosynthetic function (Zuluaga et al., 2017; Qiao et al., 2018; Figure 2).

### miR393

miR393 targets transcripts encoding the bHLH TF (bHLH77), and the auxin receptors transport TIR1 (inhibitor response protein 1), AFB1, AFB2, and AFB3 (Jones-Rhoades and Bartel, 2004; Vidal et al., 2010; Chen et al., 2011). In durum wheat, miR393c is downregulated, and the inverse correlation for miRNA393/*AFB2* has been observed in roots, leaves, and stems (Zuluaga et al., 2017). Another module, miR393/*AFB3*, also contributes to plant developmental plasticity in response to N stress via lateral root initiation and primary root elongation (Vidal et al., 2010; Tang et al., 2018; Figure 2).

### miR399

The expression of miR399s is downregulated in the roots and/or grains of wheat (Sinha et al., 2015; Hou et al., 2020), durum wheat (Zuluaga et al., 2017), and *Arabidopsis* (Liang et al., 2012) upon N starvation. However, in the roots of rice, miR399i is upregulated by N stress and has been identified as a regulator of plant nutrient homeostasis (Cai et al., 2012). In maize, miR399 is repressed in response to chronic low NO_3_^–^ conditions, while it is upregulated in response to transient low N stress (Xu et al., 2011).

Under N stress, the downregulated miR399 shows an inverse correlation with its target *PHO2* gene (Zuluaga et al., 2017), which encodes a ubiquitin-conjugating enzyme and is involved in the protein degradation pathway (Cai et al., 2012). This miRNA/target interaction may enhance proteasome-mediated N remobilization of putative targets, such as Rubisco, and play an important role in the adaptation of durum wheat to a low-N environment (Zuluaga et al., 2017).

### miRNA408

In root tissues of wheat, miR408 is differentially expressed in response to N availability (Sinha et al., 2015). Upon N starvation, the expression of miR408 is repressed in the roots/shoots of maize (Xu et al., 2011; Yang et al., 2019b) and *Arabidopsis* (Liang et al., 2012; Zhao et al., 2012). GO analysis revealed that miR408 may be involved in the regulation of N metabolism; in addition, decreased expression of this microRNA is also involved in ROS scavenging through its target gene encoding SOD1A (Yang et al., 2019b). In maize, miR408/b is preferentially expressed in root tips, epidermal cells of primary roots, and lateral root primordia (Trevisan et al., 2012a,b). These authors proposed that the repression of the transcription of miR408/b is crucial for integrating nitrate signals into developmental regulation and the adjustment of root architecture; during this process, ROS signaling may mediate this transduction pathway in response to an N shortage (Figure 2).

### miR444

miR444 is a critical regulator mediating plant tolerance to low-N stress. In wheat roots and/or leaves, miR444 is upregulated, whereas the transcription of their target genes, such as the *MIKC-type MADS-box TFs WM32B* and *WM30*, shows the opposite expression patterns in the above tissues (Zhao et al., 2015; Gao et al., 2016; Figure 2). In particular, TamiR444a transcriptionally regulates a large number of genes, including *NRT1.1-s*, *NET1.1-t*, *NRT2.1*, and antioxidant enzyme-encoding genes, including *CAT1;1*, *POD1;3*, and *POD4*, thus establishing a complicated gene network, which is associated with signaling perception and transduction, N acquisition through the modulation of NRT genes, cellular ROS homeostasis, phytohormone response, and plant growth (Gao et al., 2016; Figure 2).

However, in durum wheat, the upregulation of miR444 in response to low N is only observed in roots of one of two experimental varieties (Zuluaga et al., 2017). In rice, miR444 is only slightly upregulated by N starvation, but the levels of its three targets (MADS23, MADS27a, and MADS57) are decreased significantly (Yan et al., 2014). These authors suggested that miR444 modulates N homeostasis by mediating ANR1-like MADS-box FTs; however, they then found that upregulation of miR444a reduced the adaptability of rice plants to N-limiting conditions by reducing N remobilization from old leaves to young leaves (Yan et al., 2014; Figure 2). Moreover, the root-specific expression of osa-miR444a.4-3p is downregulated in response to N starvation, and *OsMADS25* (a novel target gene) has been shown to be targeted by osa-miR444a.4-3p; therefore, the miR444a/*MADS25* module may be involved in N acquisition and homeostasis in roots (Shin et al., 2018). Considering these conflicting results, we have to mention the most recent findings in rice roots in which NH_4_^+^ promotes the biosynthesis of brassinosteroid (BR) through miR444 to regulate rice root growth. During this process, miR444 positively regulates BR biosynthesis through its MADS-box targets, resulting in decreased root elongation (Jiao et al., 2020).

### miR827

Generally, miR827 is downregulated in roots under N starvation in durum wheat (ttu-miR827a), *Arabidopsis*, and maize (Liang et al., 2012; Zhao et al., 2012; Zuluaga et al., 2017; Yang et al., 2019b). In contrast to miR827 under low-N conditions, the expression of its target *NLA* gene increases in *Arabidopsis* (Pant et al., 2009; Liang et al., 2012) and maize (Yang et al., 2019b). GO analysis revealed that miR827 may be involved in the regulation of N metabolism by regulating the expression of the *NLA* gene, which encodes a RING-type ubiquitin ligase and has been identified as an essential component in adaptive responses to N limitation, as confirmed using an *NLA* mutation (Peng et al., 2007; Yang et al., 2019b; Figure 2).

Conversely, in the shoots or roots of oilseed rape, the expression of miR827 is upregulated by N limitations, opposite to the expression of *NLA1s* (Zhang et al., 2018). Further study showed that the miR827-*NLA1*-*NRT1.7* regulatory circuit functions as a pivotal pathway involving the adaptive responses of plants to N limitations (Zhang et al., 2018). NLA negatively regulates NRT1.7 through the protein ubiquitination pathway to meet the N requirements of plants (Liu et al., 2017). In the leaves of maize, miR827 is downregulated by short-term N deficiency but upregulated under long-term N deficiency (Xu et al., 2011). The expression of miR827 is upregulated at 12 h after low-N treatment, followed by a trend to decrease; thus, the predicted target gene *CLP* (ATP-dependent Clp protease ATP-binding subunit) shows an increase in expression at 48 h (Zuluaga et al., 2018).

In addition, a large number of miRNAs that mediate N metabolism pathways in other plants, such as miR854 (regulating ethylene-responsive transcription factor), miR2630, and miR1074 (both LRR receptor-like serine/threonine-protein kinase), have not been investigated thus far in wheat.

## N Uptake, Transport, Assimilation, and Remobilization

### N Uptake, Transport, and Related Modulators

Plants have two types of nitrate uptake systems: a low-affinity transport system (LATS) when NO_3_^–^ availability is > 0.5 mM and a high-affinity transport system (HATS) when external NO_3_^–^ availability is < 0.5 mM (Wang et al., 2012). Under low-N conditions, plants trigger constitutive and inducible expression of HATS to promote N uptake (Wang et al., 2012). In wheat, a large number of NRT and AMT (encoding ammonium transporter) genes are orthologous to those of *Arabidopsis*, barley, maize, and rice (Bajgain et al., 2018). *TaNRT1*, *TaNRT2* (Guo et al., 2014), and *TaAMTs* (Li et al., 2017) are closely associated with N starvation tolerance. TaNRT2.1 plays a major role in NO_3_^–^ uptake (Taulemesse et al., 2015). The expression of the *TaNRT2.1* gene is invariably upregulated at the early stage (14 d) of NO_3_^–^ starvation; however, it is subsequently reduced (Sinha et al., 2020). Similarly, transcription of the TaNRT2.2 gene is rapidly induced by low-N stress (Melino et al., 2015), but its level rapidly declines (Melino et al., 2018). Low N induces more accumulation of NRT2.5 and NAR2.1 (a partner protein of NRT2 proteins), which enhance N uptake, transport, and remobilization and are also involved in root growth in wheat (Guo et al., 2014; Xu et al., 2019a; Wang et al., 2020c). TaNRT1.1, TaNRT2.1, TaNRT2.2, and TaNAR2.1 are upregulated under low NO_3_^–^ conditions to increase NO_3_^–^ uptake and are closely associated with varietal differences in NUE (Jiang et al., 2017; Figure 1). In maize under N deficiency stress, the expressions of *ZmNRT2.1*, *ZmNRT2.2*, and *ZmNRT2.5* are significantly increased, and *ZmAMT1.3* and *ZmAMT3.3* do not respond to N starvation in roots and/or shoots (Dechorgnat et al., 2019). *NRT2.2*, *AMT2*, *GLNs*, *NR1*, and *NR2* are upregulated to increase N uptake and facilitate N assimilation, thereby stimulating plant growth (Ma et al., 2020). *BdNRT2* gene expression is also governed by both internal and external N status (Wang et al., 2019a). *BdNRT2/3* genes are potentially involved in HATS for root NO_3_^–^ uptake in *Brachypodium distachyon* (David et al., 2019). In the *bdnrt2.1* mutant, HATS have been shown to be reduced by 30% (Wang et al., 2019a). NRT2 is more evolutionarily conserved, and a conserved expression pattern of *NRT2* genes between species has been observed, suggesting that functional conservation may exist between species (Dechorgnat et al., 2019; Wang et al., 2019c).

The expression of NRT2/NAR genes in wheat under limited NO_3_^–^ supply is regulated by ABA signaling, contributing to the optimization of NO_3_^–^ uptake (Wang et al., 2020c). *TaNBP1*, a guanine nucleotide-binding protein subunit beta gene, improves RSA establishment, N acquisition, and cellular reactive oxygen species (ROS) homeostasis through transcriptional regulation of *NtNRT2.2* and distinctive antioxidant enzyme genes, such as *NtSOD1*, *NtSOD2*, and *NtCAT1*, resulting in a higher N-starvation tolerance for wheat (Liu et al., 2018). Basic helix–loop–helix (bHLH) TFs comprise a large TF family, act as crucial regulators in various biological processes in plants, and are involved in stress signaling. *TabHLH1*, a wheat bHLH TF member, is upregulated after N and phosphorus deprivation in roots, leaves, and/or shoots and plays an important role in transcriptionally regulating genes encoding NRTs and antioxidant enzymes that mediate cellular ROS homeostasis under nutrient stresses (Yang et al., 2016). *BIM2* (encoding a bHLH type) is highly upregulated under N stress, and alternate splicing in this gene may play an important role in N uptake through the modulation of root architecture (Subudhi et al., 2020).

NPFs (nitrate transporter 1/peptide transporters) are also transporters that are mainly involved in NO_3_^–^ uptake. In *Arabidopsis*, *AtNPF6.3* acts as a low-affinity transporter when the NO_3_^–^ level > 1 mM switches to a high-affinity mode when the nitrate level < 1 mM due to the function of CBL-interacting protein kinase (CIPK) 23 to phosphorylate intracellular threonine (Liu and Tsay, 2003). In wheat, TaNPF6.1, TaNPF6.2, and TaNPF6.3 are identified as co-orthologous to *Arabidopsis* AtNPF6.3 (Buchner and Hawkesford, 2014). Recently, alternative splicing has been considered a pattern that increases proteins and enriches the function of a gene. In rice, two variants, *OsNPF7.7-1* and *OsNPF7.7-1*, have been identified, and the expression of *OsNPF7.7s* was relatively higher in the roots or axillary buds upon low-N treatment than upon high-N treatment; altered expression of each variant could regulate shoot branching and nitrogen utilization efficiency (NUtE) and improve NO_3_^–^ or NH_4_^+^ in roots (Huang et al., 2018). However, *ZmNPF6.6* expression is downregulated, and *ZmNPF6.4*, *ZmAMT1.3*, and *ZmAMT3.3* show no response to N starvation (Dechorgnat et al., 2019).

Recently, a large number of NRT/NPF genes have been identified and are heterogeneous in terms of their gene structures and mRNA abundance (Wang et al., 2019c); in addition to NO_3_^–^ and peptides, NPFs are also involved in plant hormone transport (auxin, ABA, jasmonates, and GAs) (Wang et al., 2020b), indicating that NPF and hormone signals may act as a component of regulatory networks in response to low-N stress. Therefore, the systematic identification of gene composition, chromosomal locations, evolutionary relationships, and expression profiles contributes to a better understanding of the roles of *NPF* genes in wheat (Wang et al., 2020b).

Amino acid transporters (AATs) play an important role in amino acid uptake and remobilization from vegetative tissues to the grain. AATs are highly expressed in roots and in senescing leaves and stems, suggesting that AATs are good candidates for high NUE (Wan et al., 2017). However, the role of AATs in the wheat plant response to N starvation has not been elucidated.

As discussed above, under low-NO_3_^–^ conditions, PM H^+^-ATPase activity increases in wheat roots due to the upregulated expression of *TaHA1* (Jiang et al., 2017; Lv et al., 2021; Figure 1). In addition to the acid promotion of root growth, low-N stress has been shown to induce a greater PM H^+^-ATPase transcript abundance and increase the energy supply for N uptake (Jiang et al., 2017).

### N Assimilation

GS assimilates ammonia and glutamate to form glutamine, and ferredoxin-dependent GOGAT catalyzes the synthesis of glutamate from glutamine and 2-oxoglutarate through transamidation reactions. In wheat, low N induces the activities of both enzymes in roots and leaves, not only to promote ammonia assimilation but also to achieve osmotic homeostasis via glutamate-based synthesis of proline (Kichey et al., 2005; Yousuf et al., 2017; Lv et al., 2021). *TaNADH-GOGAT* and its interaction with *TabZIP60* play important roles in mediating N use and wheat growth (Yang et al., 2019a). The increased activities of N assimilation enzymes may be a signal for the promotion of N uptake by plant roots (Jiang et al., 2017). As discussed above, in wheat and rice, nitric oxide (NO) plays a pivotal signaling role not only in improving the N acquisition capacity and N transport by promoting lateral root initiation (Sun et al., 2015) but also in N assimilation by transcriptional and posttranscriptional regulation of the activity of N assimilation enzymes (NR, GS) under low N conditions (Balotf et al., 2018; Tang et al., 2018).

### An Important Role of Autophagy in N Remobilization

Autophagy is an evolutionarily conserved biological process in all eukaryotes for the degradation of intracellular components for nutrient (mainly N) recycling, thereby playing an important role in N remobilization and seed filling (Guiboileau et al., 2012).

In wheat, *TaATG4a* (an autophagy-related gene) shows higher transcript abundance under N deficiency (Pei et al., 2014). N starvation has been shown to enhance the expression of most of the autophagy genes tested and is involved in the regulation of N metabolism (Bedu et al., 2020), suggesting that autophagy is regulated at the transcriptomic level by N status signals. In maize, N deficiency-induced autophagy is critical during N stress and severely impacts productivity, while *atg12* mutants show reduced grain yield because of impaired N recycling (Li et al., 2015a). In foxtail millet, *SiATG8a* is localized in the membrane and cytoplasm, and its transcriptional level is dramatically induced by N starvation (Li et al., 2015b). *SiATG8a* transgenic plants have shown larger root and leaf areas and accumulated more total N than wild-type plants under N starvation conditions (Li et al., 2015b). The expression of *OsATG8a*, *OsATG8b*, and *OsATG8c* is significantly increased under N-deficient conditions and therefore enhances the activity of autophagy, N uptake, NUE, N uptake efficiency, and yield in rice and Arabidopsis (Xia et al., 2012; Yu et al., 2019; Zhen et al., 2019a,b; Fan et al., 2020). Increasing the expression of the *ATG8*, AtAtg8f, and *MdATG8i* genes in Arabidopsis increases autophagosome number; promotes autophagy activity, N remobilization efficiency, and grain filling; and confers tolerance to N limitation (Slavikova et al., 2008; Wang et al., 2016; Chen et al., 2019). Based on these findings, *ATG8* is considered a great candidate gene to increase NUE and grain yield in cereals (Yu et al., 2019).

In addition, overexpression of *MdATG18a* upregulates NRT2.1/2.4/2.5 and NIA2 genes and NO_3_^–^ uptake and assimilation and therefore enhances N deficiency tolerance and plant growth in both *Arabidopsis* and apple (Sun et al., 2018c). To date, no published study has reported that *ATG8s* are directly related to low-N tolerance in wheat; however, Yue et al. (2018) observed that the *TaAtg8* subfamily plays a crucial role in wheat tissue autophagy and stress defense, indicating that *TaAtg8* may possibly function in response to low-N stress in wheat. Regardless, it has been demonstrated in wheat that autophagy is involved in the regulation of low-N-induced promotion of root growth (Xu et al., 2019a), likely due to the regulation of oxidative stress (Masclaux-Daubresse, 2016) and N remobilization from shoots to roots.

Hormone signaling may be involved in N starvation-induced autophagy (Masclaux-Daubresse, 2016). *TaATG* promoter regions contain putative IAA and ARF elements and abiotic stress-related transcription elements, implying cross talk between autophagy and hormonal signaling, and the interactions between stresses and hormone signaling may coordinately regulate *TaAtg* gene expression in plants upon stress response (Yue et al., 2018; Liao and Bassham, 2020). In tomato (*Solanum lycopersicum*), the transcription of ATGs and the formation of autophagosomes are triggered by enhanced levels of BRs and involve BR-activated TF brassinazole-resistant1 (BZR1), which in turn enhances the degradation of denatured and unfolded proteins by autophagy, suggesting that BZR1-dependent BR signaling upregulates the expression of *ATGs* and autophagosome formation, which plays a critical role in the plant response to N starvation (Wang et al., 2019d).

## Signaling Regulation of RSA Optimization in Response to N Starvation

Plant roots are the first organ to sense the external nutrient status and therefore can immediately respond to N starvation signaling, developing an optimized RSA to enhance the acquisition of limited N nutrients (Sinha et al., 2015). The total root size consistently increased in all wheat genotypes that were examined due to nitrate (NO_3_^–^) starvation, although the shoot fresh weight decreased (Sinha et al., 2015, 2020). In wheat, the number of lateral roots, total root area, and root fresh mass significantly increased, root diameter significantly decreased, and the shoot fresh weight showed no difference at 48 h of exposure to low-N treatment (Lv et al., 2021). Interestingly, low NO_3_^–^ had a greater influence on RSA development than low NH_4_^+^ (Lv et al., 2021). In maize under long-term N stress (40 d), the length and surface area of the primary root and the average length of the seminal root-derived lateral root increased, although the whole root fresh weight decreased (Ma et al., 2020). In Arabidopsis, induction of lateral root growth by N deprivation is a “foraging” mechanism used by the plant to capture more N (Gruber et al., 2013). Therefore, optimized RSA is undoubtedly considered a morphological response of plants to N deficiency; however, to date, the detailed mechanisms by which plants perceive and transduce N stress signaling and form morphological adaptations remain largely unknown in wheat (Xu et al., 2019a).

### ROS, Protein Kinases/Phosphatases, and NO

A number of studies on wheat, rice, and maize have revealed that N starvation rapidly induces an ROS burst, which in turn functions as a component of plant signal transduction cascades in response to N limitation to induce antioxidant defense systems and adjust root architecture, thus facilitating the exploration of new regions of the soil to find additional nutrient elements (Lian et al., 2006; Trevisan et al., 2012a; Hsieh et al., 2018; Singh et al., 2018; Figure 1). However, with increasing time of N-starvation stress, the ROS content in plant tissues is negatively associated with tolerance to low-N stress (Tang et al., 2018), and the ROS burst triggers the expression of numerous genes to construct a transduction pathway, including increases in the gene expression of antioxidant enzymes, such as superoxide dismutase (SOD), peroxidase (POD), and CAT (Shin et al., 2005; Wang et al., 2019b; Yang et al., 2019b). Interestingly, miR398 and miR408, which both target [Cu-Zn]4AP/SOD, are downregulated after N stress, implying that the ROS signal and homeostasis due to the regulation of antioxidant enzyme expression might adapt to N stress (Trevisan et al., 2012a; Yang et al., 2019b). Similarly, in wheat, miR398 is downregulated under K deficiency and regulates SOD activity (Zhao et al., 2020). Additionally, in wheat, biosynthesis inhibition of miR398 in roots increases SOD activity in response to oxidative toxicity (Li et al., 2020). Increased antioxidants, such as glutathione and ascorbic acid, contribute to ROS homeostasis to maintain root development in barley (*Hordeum vulgare*); however, ROS are elevated in shoots, leading to decreased shoot dry biomass (Kováčik et al., 2014).

Protein kinases and phosphatases are well-known regulatory proteins involved in various signal transduction pathways. Many genes for protein kinases and phosphatases are upregulated or downregulated by low-N stress (Lian et al., 2006). In particular, the MAPK cascade is an evolutionarily conserved signal transduction module in plants. The MAPK signaling pathway is involved in the wheat plant response to low-N stress (Wen et al., 2015; Sultana et al., 2020; Figure 1). *TaMPK14*, a MAPK family gene in wheat, is characterized for its role in mediating the N starvation response. Compared with the wild type, the *TaMPK14*-overexpressing lines display higher antioxidant enzyme activities, improved cellular ROS homeostasis, and N accumulation under N deficiency, indicating the crucial roles of the MAPK gene in mediating the N starvation response (Shi et al., 2020; Figure 1). In durum wheat, a large number of protein kinases are differentially regulated by N starvation during grain filling, mainly including kinases functioning in MAPK cascades, tyrosine kinase-like kinases and those carrying leucine-rich repeat (LRR) domains, PKA–PKG–PKC, calcium- and calmodulin-regulated kinases, and CMGC (cyclin-dependent kinases, mitogen-activated protein kinases, glycogen synthase kinase, and cyclin-dependent kinases) (Curci et al., 2017). Consequently, critical kinases and phosphatases that are involved in the hormone signaling pathway and regulate RSA plasticity and the expression of NRTs, AMTs, N assimilation, and autophagy need to be identified.

NO is a small redox signaling molecule in plant cells that regulates plant responses to biotic and abiotic stresses, even though the complete scenario of NO generation in plants is still elusive. High homogeneity is observed during changes in ROS and NO; namely, both are simultaneously elevated in shoots, and both show no changes in the roots of barley (Kováčik et al., 2014). In *Arabidopsis*, NO production is promoted by ROS via the enhanced accumulation of mitogen-activated protein kinase (MPK) 6 during lateral root development, and *NIA2* (encoding one nitrate reductase (NR) isoform) is required for the MAPK6-mediated production of NO (Wang et al., 2010). In this process, NIA2 interacts directly with MPK6 and can be phosphorylated by MPK6, thus leading to an increase in NR activity and NO production and thereby regulating root development (Wang et al., 2010). Furthermore, in rice, N deficiency-induced NO production involves an NIA2-dependent NR pathway (Sun et al., 2015, 2016). Considering that *TaNIA2* is also upregulated under low-N stress (Mahmoud et al., 2020), and *TaMPK6* (the homologous gene of the *Arabidopsis MPK6* gene in wheat) is involved in the NR-catalyzed NO generation in wheat roots (Zhang et al., 2013), we reasonably speculate that *NIA2* may play a vital role in *MPK6* regulated NO generation under low-N stress (Figure 1).

On the other hand, PP2C9, a protein phosphatase, enhances NR activation by downregulating SnRK1 and 14-3-3 proteins, influencing N uptake and assimilation in rice under low-N stress; overexpression of *PP2C9* promotes NO biosynthesis by NR, which is activated via dephosphorylation of 14-3-3 and SnRK (Waqas et al., 2018). In wheat, WPK4, a SnRK, has been found to inactivate NR by assembling the NR and 14-3-3 complex through its phosphorylation specificity (Ikeda et al., 2000), suggesting that WPKs may be responsible for controlling N metabolism via the NO signaling pathway. As a consequence, NO generated via the NR pathway plays a pivotal role in increasing lateral root initiation, root branching, and the N uptake rate, thus improving the N acquisition capacity in wheat and rice under low-N stress (Sun et al., 2015; Balotf et al., 2018; Waqas et al., 2018; Figure 1). Considering that both NO and low NO_3_^–^ are involved in root growth modulation and are metabolically connected, it is reasonable to postulate that NO may be an important signal participating in low-N signaling and mediating plant adaptation.

A study demonstrated that low-N-induced NO in root tips are involved in cross talk with strigolactones (SLs), leading to the elongation of seminal roots via induction of meristem cell activity in rice (Sun et al., 2016). A subsequent study revealed that auxin acts as a downstream regulator of NO and SL signals to induce meristem activity in root tips in rice under low N, and *OsPIN1b*, encoding an auxin efflux protein, is involved in auxin transport (Sun et al., 2018a; Figure 1).

### Auxin

Plant hormones are important components of signal transduction for N stress (Sultana et al., 2020). A large body of studies has demonstrated the existence of interactions between N nutrition and auxin signaling and revealed the roles of auxin polar transport and signal transduction in the regulation of RSA modifications in response to N availability. Low-N conditions induce the biosynthesis of auxin in the roots of *Arabidopsis* seedlings (Kiba et al., 2011) and wheat (Lv et al., 2021). Pyridoxal phosphate-dependent transferase, an important coenzyme for catalyzing transamination of L-tryptophan for auxin biosynthesis, is upregulated in roots of durum wheat (*T. turgidum* ssp. Durum) under N starvation; accordingly, auxin-responsive factors (ARF2 and ARF18) are also upregulated in the same tissues (Curci et al., 2017). In *Arabidopsis*, the tryptophan aminotransferase-related gene (*TAR2*) is induced by low N conditions, and this gene functions in the tryptophan-dependent pathway of auxin biosynthesis and is expressed in the pericycle and the vasculature of the mature root zone near the root tip (Ma et al., 2014). In wheat, *TaTAR2.1* is upregulated by low-N availability and expressed mainly in roots. Overexpressing *TaTAR2.1-3A* enhances the auxin content in the root tip and promotes lateral root branching and shoot N accumulation (Shao et al., 2017). These results suggest that the interaction between *TAR2* and auxin, as well as enhanced auxin biosynthesis, is required for modifying RSA in response to low-N conditions.

In maize and Arabidopsis, low-N availability enhances shoot-to-root auxin transport and auxin accumulation, which induces lateral root growth and RSA development via auxin-dependent acid growth and the auxin-regulated target of the rapamycin pathway (Tian et al., 2008; Asim et al., 2020; Sun et al., 2020b). *OsPIN1b*, an auxin efflux carrier gene, is significantly downregulated in rice root under low-N stress (Sun et al., 2018a). In maize, *ZmPINY* is also downregulated, and the *ZmLAX4* auxin influx carrier gene is induced in roots by N starvation; accordingly, a large proportion of the downstream auxin response genes are upregulated, implying that auxin polar transport and flux may play an important role in the reprogramming of the root architecture of plants under N deficiency (Ma et al., 2020). In wheat, expression of *TaPIN1*, *TaPIN2*, *TaPIN3*, and *TaPIN4* is reduced in seedlings under low-Pi availability, thus stimulating lateral root initiation (Talboys et al., 2014). Assay using a non-invasive micro-test technology showed that the rate of IAA influx at the root surface significantly increases at early hours of low-N stress; as a consequence, the enhanced root IAA concentration stimulates the root branching and elongation (Lv et al., 2021). However, the roles of *TaPINs* and *LAX* genes in wheat response to low-N stress remain to be elucidated.

In contrast, Maghiaoui et al. (2020) proposed that low-NO_3_^–^ stress represses *LAX3* and *TAR2* gene expression through NRT1.1, which acts as a negative regulator of the auxin biosynthetic gene expression, thus preventing the growth of lateral root primordia. However, researches are needed to understand how N affects the underlying auxin signaling processes in governing RSA, especially in wheat.

Under low-N conditions, significantly increased lateral root growth is accompanied by enhanced expression of plasma membrane (PM) H^+^-ATPase AHA2 (Młodzińska et al., 2015). In this scenario, H^+^-ATPase activity is probably regulated by endogenous auxin transport because treatment with 2,3,5-triiodobenzoic acid, an inhibitor of the polar transport of auxin, significantly decreases the H^+^-ATPase activity in the root cells and inhibits low-N-induced lateral root initiation and growth (Sun et al., 2020b; Lv et al., 2021), suggesting that the increased auxin level may enhance root elongation by acidifying the root cell wall and enhancing cell elongation in response to the low-N supply, which is crucial for the development of the root architecture and the plant to explore a greater space for N resources.

NRT1.1, a nitrate transporter, has a role in signaling and promotes auxin transport from the lateral root tip toward the shoot to prevent auxin accumulation in lateral root primordia and young lateral roots under low NO_3_^–^ levels (Zhang et al., 2007; Gojon et al., 2011), thus reducing lateral root development (Krouk et al., 2010). In contrast, NRT2.1, a high-affinity nitrate transporter, facilitates NO_3_^–^ transport across the plasma membrane and acts as a positive regulator of lateral root initiation under low-N conditions in *Arabidopsis* (Remans et al., 2006). Whether this process is related to auxin polar transport is still unknown. Additionally, Loqué et al. (2006) observed that the ammonia transporter gene *AMT1.1* and *AMT1;3* play a critical role in restructuring lateral root architecture in *Arabidopsis* under N starvation.

### Abscisic Acid

A low NO_3_^–^ (< 1.0 mM) supply stimulates lateral root elongation, while a high level of NO_3_^–^ inhibits root development, where ABA plays an important role in mediating the effects of NO_3_^–^ on lateral root formation (Signora et al., 2001). Wheat zeaxanthin epoxidase, an important ABA synthesis-related enzyme, is significantly upregulated, and the ABA contents are significantly enhanced in the roots and leaves of wheat under low-N stress, suggesting a role of ABA in the tolerance of plants to N deficiency (Kang et al., 2019; Mahmoud et al., 2020). Kang et al. (2019) proposed a putative ABA-dependent schematic model in higher plants under N deficiency. Plant cells can perceive external N deficiency in the cell membrane, and this signaling is subsequently transduced by Ca^2+^ sensors, kinases, or other signaling components, which then induce translational changes in many functional proteins related to ABA. ABA and other phytohormones then regulate downstream posttranscriptional responses, followed by morphological and physiological adaptive changes that assist survival under N-starvation stress (Kang et al., 2019; Figure 1). However, Ma et al. (2020) reported that all ABA-related genes are downregulated in maize under low-N stress. ABA receptor PYL2 is also downregulated by low-N treatment in roots, which is accompanied by longer primary roots (Xu et al., 2019a).

In rice, RPN10 (26S proteasome regulatory particle non-ATPase subunit 10) regulates plant sensitivity to both auxin and ABA, which is repressed by low-N treatment (Ding et al., 2011), whereas the expression of key TF genes for ABA and auxin is rapidly induced by N starvation (Hsieh et al., 2018). These results suggest that the interactions between phytohormone signals may be involved in regulating the responses to low-N stress. A persuasive argument has been proposed that the interactions between auxin and ABA induce cell division and elongation and thereby promote primary root growth (Hsieh et al., 2018; Figure 1).

### Cytokinins, GAs, SA, and JA

Cytokinins are also closely associated with N deficiency signaling, act as both local and systemic signals, and participate in the regulation of RSA modifications and N acquisition under N-deficient conditions (Kiba et al., 2011; Lv et al., 2021). Two genes encoding cytokinin dehydrogenase are significantly downregulated by N deficiency as a strategic response to N deficiency stress (Shao et al., 2020). Adenosine kinase plays an important role in the conversion of cytokinins toward nucleotides (Kwade et al., 2005). In response to low-N stress, adenosine kinase is downregulated, suggesting that adenosine kinase may be involved in cytokinin signaling to regulate low-N tolerance (Ding et al., 2011).

Recently, Xiong et al. (2019) observed that the mutation of gibberellin (GA)-responsive protein genes results in resistance to low-N stress in wheat. The level of GA_3_ is significantly increased under low-N stress (Lv et al., 2021). Most notably, low-N nutrition promotes the expression of the *AtNPF3.1* gene, which is involved in GA transport in plants under low-nitrate conditions (David et al., 2016). Eight genes related to GA synthesis are differentially expressed under N starvation (Ma et al., 2020). In addition to these hormones, JA and SA are also implicated in the response to low-N conditions (Lv et al., 2021; Sun et al., 2020a). JA signaling may function by regulating sugar biosynthesis, amino acid biosynthesis, and absorption of NH_4_^+^ (Sun et al., 2020a).

Taken together, most hormones, such as GAs, ABA, and cytokinins, may be involved in the regulation of root growth and development, likely through crosstalk with auxin (Ma et al., 2020).

## Concluding Remarks and Future Perspectives

First, it is imperative to identify the cell membrane and intracellular receptors that perceive N starvation and downstream signals and to investigate how these signals are transduced to gene expression and protein translation and then integrated with pathways to regulate root growth and development. At the transcriptional level, the exact functions of the differentially expressed genes and the timing and tissue specificity of the transcriptional responses to low-N stress are most important for cereal crops with high NUE under suboptimal N conditions. Thus, employing a combination of multiomics-assisted analyses, such as SWATH-MS-based proteomics, proteogenomics, and *de novo* peptide sequencing, is useful for deciphering low-N signaling, screen downstream signaling components, and generate metabolic pathways and networks to better understand the regulatory mechanisms at developmental, molecular, and physiological levels. The identification of central members, such as the regulatory circuit miR827-NLA-NRTs, in the network will facilitate our understanding of the molecular mechanisms underlying the plant response to external N signaling mediated by miRNA/target pathways. The plant signaling system is very important for plant breeders and agronomists and enables them to improve NUE.

The generation of transgenic lines based on valuable gene resources, such as *TAR2.1*, *NBP1*, *bZIP60*, and *NADH-GOGAT*, is critical for the development of large RSAs, improving N uptake and assimilation, and shows potential for genetic engineering to increase the grain yield of wheat under N-saving cultivation conditions. Transcription factors may be a potential gene for molecular breeding and cultivation of wheat to improve N remobilization, utilization, and yield in crops simultaneously. Genetic programing through temperature- or photoperiod-controlled timing expression of genes, such as *ATGs*, would promote N remobilization during grain filling and increase NUE.

In recent years, genome editing with engineered nucleases has been used in plant breeding. Further identification of the genes that negatively control NUE and agronomically important traits will facilitate the use of genome editing to increase NUE and yield. These large-scale datasets provide valuable information for the generation of new wheat cultivars with higher NUE or greater resistance to N starvation in future breeding programs.

In conclusion, many links between signal perception, transduction, and regulation of gene functions at the transcriptional, posttranscriptional, and translational levels are still missing. Although we have filled the putative gaps with knowledge translated from other plants, a genetics-based approach is needed to find molecular targets in the field of N starvation, which is an effective way to screen signaling components and to construct regulatory networks in response to N starvation.

## Author Contributions

LK drafted the manuscript. YZ and WD searched the literature and provided suggestions for writing. HX and SF modified the manuscript. BZ conceived the project, constructed figures, and gave suggestions on the revision of the manuscript. All authors contributed to the article and approved the submitted version.

## Conflict of Interest

The authors declare that the research was conducted in the absence of any commercial or financial relationships that could be construed as a potential conflict of interest.

## Appendix

**APPENDIX TABLE 1:** Signaling or functions of differentially expressed miRNA families in response to low-N stress.

miRNA	Potential target	Species/tissues (expression)			Function annotation	References	
		Wheat	Rice	Maize	Arabidopsis		
**Hormone signal-related**							
miR1133	bZIP TF superfamily protein; SET domain, early nodulin proteins	Wheat root (down)		Maize seedling (down)		Involved in ABA signaling; increasing NADH-GOGAT activity; lateral root branching; N uptake	Zhao et al., 2015; Yang et al., 2019b
miR156	Squamosa promoter-binding-like protein	Wheat root (up)	Rice leaf (down)			Auxin signaling to modulate lateral root growth and anthocyanin biosynthesis	Nischal et al., 2012; Zhao et al., 2015
miR159	GAMYBs, MYB, TCP transcription factors; MYB3, MYB33, MYB65	Wheat grain (down), durum wheat leaf and root (down)	Rice root (down for -NO_3_^–^, up for -NH_4_^+^)	Maize root (up)		Associated with GA signaling; plant development	Zhao et al., 2012; Sinha et al., 2015; Li et al., 2016; Shin et al., 2018; Zuluaga et al., 2018; Hou et al., 2020
miR160	*ARF8*, *ARF10*, *ARF16*, *ARF17*, *ARF18*, and *ARF22*	Wheat root (down)	Rice root (down)	Maize root (up)	*Arabidopsis* (up)	Reducing auxin-responsive activities and vegetative growth	Xu et al., 2011; Liang et al., 2012; Shin et al., 2018; Zuluaga et al., 2018
miR164	*NAC*; *NAM*	Wheat (down), durum wheat root (up)	Rice root (up or down)	Maize leaf (up)		Mediating auxin-induced lateral root development; accelerating N remobilization; involved in shoot apical meristem formation	Guo et al., 2005; Xu et al., 2011; He et al., 2015; Li et al., 2016; Sinha et al., 2015; Shin et al., 2018
miR167	*ARF6*, *ARF8*	Durum wheat root (down), durum wheat leaf and stem (up)		Maize root (down)	*Arabidopsis* root (down)	Auxin-mediated plasticity of root architecture; initiation and emergence of lateral roots	Gifford et al., 2008; Xu et al., 2011; Zuluaga et al., 2017; Zuluaga and Sonnante, 2019
miR169	*NFYAs*	Wheat leaf, grain and root (down)	Rice root (down)	Maize root and leaf (down)		Involving the ABA and auxin signaling pathways; long-distance signaling of N, associated with nutrient deficiency; stress response	Xu et al., 2011; Zhao et al., 2011; Nischal et al., 2012; Trevisan et al., 2012b; Zhao et al., 2012; Shin et al., 2018; Hou et al., 2020
miR319	MYB and TCP TFs	Durum wheat root, leaf and stem (up)		Maize root (up)		Hormone biosynthesis and signal transduction to low N; root meristem growth; oxidoreductase (defense response)	Vidal et al., 2010; Xu et al., 2011; Guan et al., 2017; Zuluaga et al., 2017; Zuluaga and Sonnante, 2019; Hou et al., 2020
miR393	bHLH TF and TIR1; AFB1, AFB2, and AFB3	Wheat root (up), durum wheat root, leaf and stem (down)		Maize (up)		Auxin receptors and signaling; regulating development of primary and lateral roots defense response	Zuluaga et al., 2017, 2018; Zuluaga and Sonnante, 2019; Yang et al., 2019b
**Ca^2+^ signal-related**							
TamiR1118	*Calmodulin TaCaM2-1*	Wheat root (up)					Zhao et al., 2015
TamiR1133	Calmodulin-like protein	Wheat root (down)					Zhao et al., 2015
miR164	OsCML22 Calmodulin-related calcium sensor protein, ubiquitin family protein	No homologs with similar targets is identified	Rice root and leaf (down)			Low-N tolerance pathways and functions	Nischal et al., 2012
**ROS signal-related**							
miR1133	NADH-ubiquinone/plastoquinone oxidoreductase, chain 6 precursor	Wheat root (down)					Zhao et al., 2015
miR1214	Peroxidase 2-like; F-box domain containing protein	No homologs are identified		Maize seedling (down)		Involved in N metabolism regulation	Yang et al., 2019b
miR398	Superoxide dismutase [Cu–Zn]4AP/SOD4AP	Homologs identified involved in ROS scavenging		Maize seedling (down)	*Arabidopsis* seedling (down)	Involved in N metabolism	Liang et al., 2012; Yang et al., 2019b; Li et al., 2020; Zhao et al., 2020
miR408	Superoxide dismutase [Cu-Zn]1A/SOD1A; L-ascorbate oxidase;	Wheat root (down)		Maize root and shoot (down)	*Arabidopsis* seedling (down)	Regulating the expression of stress response factors, N metabolism and root architecture	Xu et al., 2011; Liang et al., 2012; Zhao et al., 2012; Sinha et al., 2015; Yang et al., 2019b
miR444	*MADS-box TFs*;*NtNRT1.1-s*, *NtNET1.1-t*, *NtNRT2.1*, *NtCAT1;1*, *NtPOD1;3*, and *NtPOD4*	Wheat root/leaf (up), durum wheat root (up)	Rice root (down)			Cellular ROS detoxification; increasing N acquisition	Zhao et al., 2015; Gao et al., 2016; Zuluaga et al., 2017; Zuluaga and Sonnante, 2019; Shin et al., 2018
miR528	*Cu/Zn SOD*, *POD*, L-ascorbate precursor; IAR1, CBP/OsDCL1,	Wheat grain (down)	Rice root and leaf (down)	Maize leaf (up) or maize shoot and root (down)		Enhancing ability to scavenge ROS; ROS and IAA homeostasis	Xu et al., 2011; Nischal et al., 2012; Trevisan et al., 2012b; Zhao et al., 2012; Hou et al., 2020
**Protein phosphorylation signal-related**							
miR3454	Protein kinase family protein	No homologs identified		Maize		N compound metabolic process	Yang et al., 2019b
miR156	Kinase domain	Wheat root (up), wheat grain (down)	rice leaf (down)				Hou et al., 2020; Nischal et al., 2012; Zhao et al., 2015;
miR169	MDIS1-interacting receptor like kinase 1 protein, serine/threonine kinase	Wheat grain (down)		Maize root and leaf (down)		Regulating expression of NRT2.1 and NRT1.1, N uptake and defense processes	Xu et al., 2011; Zhao et al., 2011; 2012; Yang et al., 2019b; Hou et al., 2020
miR399	*TNNI3K* encoding serine/threonine-protein kinase; genes encoding TFs, oxidoreductase and UDP-glucosyltransferase	Wheat grain (down)	Rice root (up)			Regulators of plant nutrient homeostasis	Cai et al., 2012; Hou et al., 2020
miR2275	PRP, WRK, SPK (encoding for a serine/threonine protein kinase 19)	Wheat (up)				Regulating expression of NRT2.1 and NRT2.2, increasing N acquisition and the biomass	Qiao et al., 2018
**N metabolism related**							
miR156	Clathrin adaptor complex small chain domain	Wheat root (up)	Rice leaf (down)			N metabolism, and protein/peptide degradation	Nischal et al., 2012; Zhao et al., 2015
miR399	*PHO2* (*ubiquitin-conjugating E2 enzyme*)	Wheat root and leaf (down)	Rice root (up)	maize (up)	*Arabidopsis* (down)	Protein degradation and N remobilization	Xu et al., 2011; Liang et al., 2012; Cai et al., 2012; Sinha et al., 2015; Zhao et al., 2015
miR827	*NLA*, CLP (ATP-dependent Clp protease ATP-binding subunit)	Wheat grain (down), durum wheat root (down)		maize root (down) and leaf (up)	*Arabidopsis*(down)	Regulation of N limitation adaptation response; N accumulation and remobilization by regulating NLA	Xu et al., 2011; Liang et al., 2012; Zhao et al., 2012; Zuluaga et al., 2017; Zuluaga and Sonnante, 2019; Yang et al., 2019b; Hou et al., 2020
